# Improving the surgical consenting process for patients with acute hip fractures: a pilot quality improvement project

**DOI:** 10.1186/s13037-020-00252-8

**Published:** 2020-06-13

**Authors:** Kirit Singh, Ali Assaf, Morgan Bayley, Gordon Gillespie

**Affiliations:** 1 Department of Trauma and Orthopaedics, Royal Gwent Hospital, GNG T&O Secretaries Office, Level 5, Cardiff Road, Newport, NP20 2UB Wales; 2 Department of Trauma and Orthopaedics, Withybush Hospital, Pembrokeshire, Wales

**Keywords:** Checklists, Control charts/run charts, Reminders, Surgery

## Abstract

**Background:**

Consenting patients for trauma procedures following hip fracture is a key stage in the treatment pathway from admission to the operating theatre. Errors in this process can result in delayed procedures which may negatively impact patient recovery. The aim of this project was to identify and reduce errors in our consenting process for patients with capacity.

**Methods:**

Consent forms for all adult patients with capacity admitted for surgical repair of traumatic hip fracture were reviewed over a 4-week period. The baseline measurement (*n* = 24), identified errors in three key process measures: clarity of documentation, failure to record procedure-specific risks and not offering a copy of the consent form to the patient. Pre-printed stickers and targeted teaching were then introduced as quality improvement measures. Their impact was evaluated over subsequent 4-week review of the same patient demographic, with further refinement of these interventions being carried out and re-evaluated for a final cycle.

**Results:**

Cycle 1 (*n* = 26) following targeted teaching demonstrated a reduction in abbreviations from 38 to 20%, while doubling the documentation of discussion of procedure-specific risks from 31 to 72%. More patients were offered a copy of their consent form, rising from 12 to 48%. Cycle 2 (*n* = 24) saw the introduction of pre-printed “risk of procedure” stickers. Although clarity measures continued to improve, quality of pre-procedure risk documentation remained static while the number of forms being offered to patients fell to 8%.

**Conclusions:**

Our project would suggest that while pre-printed stickers can be useful memory aids, specific teaching on consenting produces the greatest benefit. The usage of such tools should therefore be limited, as adjuncts only to specific training.

## Background

Hip fractures are one of the most common presentations to emergency departments in the United Kingdom with 66,000 cases reported per year [1]. This is a significant injury that carries a 6.9% mortality risk within 1 month and requires prompt management [1]. Delay of surgical intervention (if indicated) beyond 48 h is associated with increased death rates and complications such as pressure sores [2]. Trauma & Orthopaedics departments are therefore required to make the pathway to operation as efficient as possible to ensure that patients are operated on within this window.

A key element of this pathway is gaining the patient’s consent for the procedure. Patients should have their treatment options explained to them, along with the benefits of any intervention, along with associated risks, burdens and side effects before then reaching a decision on whether to proceed [3]. Commonly for acute trauma admissions, this consenting process is performed by the clerking doctor to prepare the patient for an imminent operation. However often the clerking doctor may not be the surgeon performing the procedure and can be a junior grade physician who is working under significant time or work pressure [4]. These pressures have been noted to result in poor documentation and missed elements of admission notes [5] which can render important documentation such as consent forms substandard or even invalid [4].

In order to reduce the variation in the admission and consenting process, several approaches have been evaluated, ranging from enhanced training to the use of procedure-specific proformas [6] and consent stickers with pre-printed risks [7]. Although these have demonstrated benefit, their usage remains limited and best practice is still unknown. This quality improvement project aimed to assess our baseline consenting practice within a large 774-bed district general hospital while also reviewing the impact of ongoing improvement efforts such as teaching sessions and the use of proformas and consent stickers.

Reviews of consent documentation for common trauma procedures have found poor documentation of risks [8] often with a wide variety of complications listed for the same procedure in varying frequencies [9]. These deficiencies in documentation have been attributed to not only the aforementioned work pressures but also poor overall awareness of local or national guidelines and standards [10], which is often exacerbated by a relatively rapid turnover of junior staff (rotating as frequently as every 4 months) [5]. This is despite Department of Health and Royal College of Surgeons guidelines on consent that state the person obtaining consent should have clear knowledge of the procedure along with the potential risks and complications [11, 12].

Poor or incomplete documentation during the consent process denies the patient a full understanding of the procedure they are about to undergo, negatively impacting their decision making autonomy [13]. This can also then subsequently expose hospitals and doctors to possible litigation [14, 15]. Previous reviews of litigation relating to informed consent in the United States found errors in recording the risks of a procedure accounted for up to 70% of complaints alone [16]. Furthermore, if consent documentation is of poor enough quality to be rendered entirely invalid, this can delay the operative pathway, potentially resulting in cancellation of procedures [4]. In a time-critical situation such as hip fractures, these unnecessary delays can therefore significantly negatively impact patient care and outcomes.

Set proformas/checklists for common presentations have been found to improve the documentation of important items which can commonly be missed [17]. Checklists of essential pre-operative tasks have been used to improve the rate of completion of requested jobs [18] and their usage in surgical ward rounds has also been found to improve the quality of documentation [19]. It has also been established that checklists can have a significant role in changing cultural practice and improving safety [20] with work by the Safe Surgery Saves Lives Study Group finding that a checklist was associated with concomitant reductions in both rates of death and complications in patients undergoing non-cardiac surgery [21].

Stickers have been used as part of a checklist, for documentation of ward rounds and demonstrated to be a simple and effective way of evidencing good practice against recommended standards [22]. A quality improvement program in NHS Lanarkshire where the use of ward round stickers was audited found them to be a simple and effective safeguard to ensure basic aspects of care were not missed while maintaining standards [23]. The benefits of this standardized approach subsequently led to the development of websites devoted to consent for specific procedures. The goal of these resources was to provide a uniform approach to documenting risks, and their use has been shown to produce an improvement in quality of consenting [24]. Similar benefits were found when using pre-printed consent labels for consenting patients [7, 25].

However, these efforts are prone to abandonment when local champions of their use move on [26] and it is recognized that the usage of proformas often tails off over time for a variety of reasons (e.g. logistical issues with re-printing when initial supplies are depleted) [27]. This Quality Improvement project therefore sought to determine whether targeting teaching and/or pre-printed consent stickers would demonstrate sustained improvements in quality of consent.

## Method

### Baseline measurement

Over a 4-week period in August 2017, consent forms for patients admitted as emergency trauma due to hip fracture were audited against Royal College of Surgeons Standards [28]. Patients were included if deemed to have mental capacity to consent for a procedure and excluded if they did not. This was achieved by only selecting consent form documentation that was used for those people aged 16 and over and having capacity, or for those under 16 years who were determined to be Gillick competent (referred to locally as Consent Form 1 [29]). All other consent form types were not reviewed for the purposes of this project (such as those for paediatric cases or for those lacking capacity.)

All data on the forms were analysed, with special attention being given to three process measures of clarity of documentation, whether the listed risks were appropriate for the intervention and if a copy of the consent form had been given or offered to the patient. Evaluation of clarity focussed on if all the patient details had been filled in correctly, along with if the procedure details were correct including laterality and site of surgery and whether abbreviations had been used.

In this period, 24 consent forms for neck of femur fracture patients with capacity were identified. While 93% had accurate completion of patient details and documentation of the patient’s agreement to consent, 38% of forms used abbreviations instead of fully writing out the procedure. Documentation of generic risks such as infection, bleeding and thromboembolic events was completed in 95% of reviewed forms. However, significant procedure-specific risks such as dislocation and leg length discrepancy were documented in just 31% of forms. Finally, only 12% of patients were offered or given a copy of their consent form.

### Design

Given the significant omissions in consent form documentation and the consenting process, it was clear that improvement was necessary. The majority of documentation had been completed by junior members of the team, over a period that represented their first 4 weeks within the speciality. It therefore was felt that to improve documentation of procedure-specific risks, further teaching would be necessary to improve their understanding of the operation and what significant complications could arise. Improved oversight was also to be implemented with ongoing senior clinician review of documentation to be performed on the morning ward round to identify any errors (such as abbreviations) or omissions. Recognizing also that junior staff were completing these documents often in time or work pressured environments, further memory aids were also reviewed and developed including pre-printed consent form stickers with all key procedure-specific risks included. These could then be directly applied to the generic consent forms that were currently in use.

### Strategy and improvement cycles

Having determined that lack of experience in junior trainees was a key reason for poor documentation, interventions were planned to align with each rotation where new trainees would start working within the department. Practice would then be re-audited over another four-week period to determine the impact of any interventions made.

Improvement Cycle 1 (January 2018) – Teaching for junior colleagues was carried out, including a session at induction for newly rotated junior doctors on how to complete consent in accordance with Royal College of Surgeons guidelines [12]. Other teaching sessions were also performed for the department as a whole, covering the most common risks of procedures performed for high frequency presentations. Alongside this, senior review of consent forms in the morning ward round to identify errors was implemented. However, errors still occurred with procedure-specific risks found to be absent in 28% of cases.

**Fig. 1:**
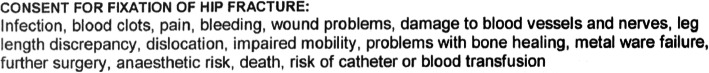
Consent Sticker Scan

Improvement Cycle 2 (August 2018) – As part of an updated approach to managing hip fractures, a proforma was developed including pre-printed consent stickers with all risks (both generic and procedure-specific) included (example for intracapsular neck of femur fractures requiring arthroplasty shown in Fig. 1). These stickers were then to be used in conjunction with existing consent form documentation and act as a memory prompt for the doctor’s discussions with the patient. A teaching session on the use of this form along with the key procedure-specific risks was given to junior staff at their induction as they began their rotation.

## Summary of results

Following the teaching interventions in cycle 1, re-audit of practice identified 26 consent forms suitable for analysis. Significant improvement was seen in reducing the usage of abbreviations, which fell from 38 to 20% and procedure detail documentation improved further from a baseline of 93 to 96%. Improvement was also found in documentation of all risks improving from 66 to 81% and procedure-specific risks documented in 72% of analysed forms, more than doubling baseline measurements. More patients were also offered a copy of their consent forms, rising from 12 to 48%.

**Fig. 2:**
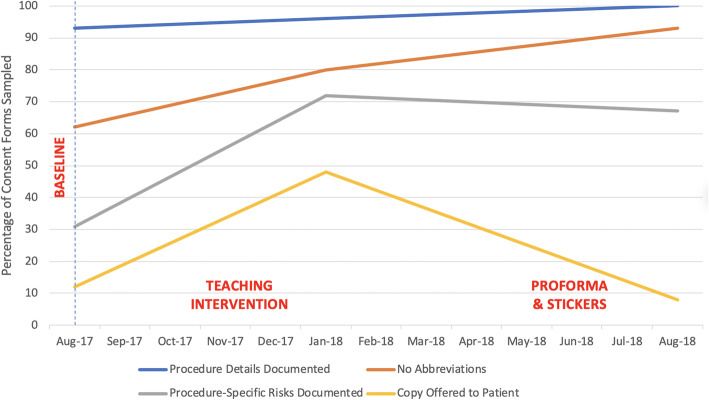
Runchart of Improvement Cycles

With the introduction of the hip fracture proforma and consent stickers in cycle 2, a further 24 consent forms were evaluated (see Fig. 2 – summary runchart). Sustained improvements were seen in documentation of procedure details, rising to 100% completion on all forms sampled. Abbreviation usage continued to fall further, decreasing to 7%. However, documentation of procedure-specific risks remained similar to the prior cycle at 67% while a significant deterioration was seen in patients being offered a copy of their consent form, falling to just 8% of patients. Raw data for all aforementioned quality outcome indicators is presented in Table 1.

**Table 1:** Quality outcome indicators

	Baseline	Cycle 1	Cycle 2
**Procedure details documented**	93	96	100
**No abbreviations**	62	80	93
**Procedure-specific risks documented**	31	72	67
**Copy offered to patient**	12	48	8

## Discussion

While improvements in consenting practice were seen and maintained, it is notable that targeted interventions like stickers for risk documentation failed to produce significant change, with other measures such as copies being offered to patients slipping back. However, this analysis was limited by issues relating to the deployment of such pre-printed proformas and stickers. As mentioned previously, these interventions can be prone to attrition over time, and it was reported that supplies of the stickers were exhausted quickly with replacements reported as difficult to locate by junior colleagues, limiting their usage. Records audited later in cycle 2 showed their formal use fell to less than 75% of the consent forms evaluated. However, doctors within the team reported to the authors that a photo stored on their smartphone was used for reference when manually writing out the consent documentation, indicating ongoing usage of the pre-printed information by proxy. Furthermore, training on their content had also been delivered at time of the intervention, so the audit cycle was continued.

Further, as this project only focussed on patients with capacity, areas of poor practice may persist for those who do not. Given the demographic of patients admitted with hip fracture, many patients have co-morbidities that can impair their mental capacity, either of irreversible aetiology such as dementia, or temporary such as delirium. Documentation of this process is longer and more time consuming, with the documentation used locally being several pages [29]. Given that a formal assessment of capacity needs to be documented as well as any discussions (including procedure-specific risks) with family members or concerned parties, this is likely prone to the same pressures and issues causing poor practice outlined previously. Future work within the department will seek to review this documentation as well as considering specific interventions of benefit here.

Nevertheless, our experience from this improvement project finds that teaching sessions focussed on consenting and complications provided the greatest benefit, as well as developing newly rotated doctors understanding of the speciality. This would correspond as well with the recent recognition of the need for a personalised approach for consenting (as per Montgomery vs NHS Lanarkshire [30]). Further, the teaching sessions seemingly generated ongoing cultural change in the department, with the usage of abbreviations continuing to decline without further input. From the literature, it has been demonstrated that pre-printed stickers and proformas can generate benefit and in our study, it would seem that some previous benefit was sustained with their introduction. Given the experiences within this quality improvement project where physical sticker supplies were quickly depleted, but doctors used photos of the pre-printed stickers stored on their smartphones, apps may also provide a less resource intensive method of achieving the same objective. This may also prove more cost-effective in the long term given that beyond the initial development cost, there would be no ongoing need to reprint and restock such items. Such a cost-benefit analysis was beyond the scope of this pilot study but will be considered in future work. However, it is notable that copies of forms being offered to patients declined significantly after the initial teaching session, which was not prompted for in the new hip fracture proforma. This suggests that such approaches, especially in the context of a busy admissions environment, can engender a degree of automaticity by the consenting physician, with other elements of the clerking and documentation process being neglected.

While further prompts within proformas may help to improve this, ultimately it is impossible to checklist the whole process and so these cannot serve to replace high quality training. Instead it would suggest that the role of proformas be limited and only be rolled out alongside detailed training on the condition they are covering. Without this, although consent forms may show thorough documentation of risks, the discussion between doctor and patient may be limited to merely reading off a list, rather than a detailed explanation. This is likely to be of increasing importance to consider as many trusts move to computerized admission records and digital consenting. As the department has now formally introduced dedicated training on the consenting process for all new doctors being inducted into the department, future work will seek to evaluate the efficacy of teaching interventions specifically with larger cycles.

## Conclusions

Errors or omission of important elements in documentation of patient consent can delay operations, expose hospital trusts to medico-legal risk and ultimately may represent a failure to fully respect patient autonomy. Our project sought to evaluate our current consenting practice for hip fractures and reduce the error rate in recognized deficiencies of clarity of documentation, documentation of procedure appropriate risks and ensuring that patients were offered a copy of their consent form. While teaching sessions for the department brought about large improvements in our key process measures, the use of pre-printed stickers failed to significantly enhance this, and sharp deterioration was seen in areas that focus was taken away from. Accordingly, the department at the pilot hospital now dedicates specific training time on consenting for common Trauma & Orthopaedics interventions for all new doctors at the start of their rotation. As digital consent and the use of pre-prepared documentation is set to expand, it is important to recognize the value of detailed training on consenting and procedure risks, so that not only is documentation of a high quality but also the patients actual understanding stemming from their conversation with the consenting doctor. This would therefore seem to require an ongoing personalized approach to consenting. Pre-printed checklists and proformas can be valuable tools to assist busy doctors, but their usage should be limited to adjuncts only and not be allowed to replace a high-quality discussion between physician and patient.

## Data Availability

All data is stored securely within National Health Service information technology infrastructure. Please contact the corresponding author for access to data.

## Abbreviation

NHS: National Health Service
